# Implications of implicit stereotypes in nursing education: A study using projective techniques in light of transformative learning theory

**DOI:** 10.1016/j.jtumed.2026.10.008

**Published:** 2026-11-04

**Authors:** Małgorzata Lesińska-Sawicka

**Affiliations:** Department of Nursing, State University of Applied Sciences in Konin, Przyjaźni 1, Konin, Poland

**Keywords:** الكفاءة الثقافية, طلبة التمريض, الصور النمطية الثقافية الضمنية, التحيز الضمني, التقنيات الإسقاطية, التأمل الذاتي, التعلم التحويلي, التعليم التمريضي, Cultural competence, Implicit cultural stereotypes, Nursing education, Nursing students, Self-reflection, Spontaneous associations

## Abstract

**Background:**

Cultural competence is a fundamental component of professional nursing practice in increasingly diverse healthcare settings. Nursing students often declare their openness and egalitarian attitudes, but unconscious cultural stereotypes may still influence perceptions and interactions with patients. These implicit cognitive patterns are difficult to capture using self-report measures alone.

**Aim:**

This study aimed to explore implicit cultural stereotypes identified in nursing students’ spontaneous associations and to examine the discrepancy between these implicit associations and explicit self-reflection on cultural competence.

**Methods:**

A mixed-methods exploratory design with a mainly qualitative component was applied. Data were collected from 33 s-cycle nursing students recruited through convenience sampling at a single academic institution, which may have limited the representativeness of the sample. Participants completed a projective association test designed to elicit spontaneous responses, followed by an open-ended self-reflection task. Qualitative thematic analysis was supported by supplementary quantitative descriptive analysis of the response distribution.

**Results:**

In total, 482 response units were analyzed. Negative stereotypes accounted for 36.1% of responses, positive stereotypes for 16.8%, and neutral statements for 47.1%. Stereotypical associations most frequently concerned patient behavior, communication, national or ethnic origin, and religion. Despite the presence of stereotypical content in spontaneous associations, explicit self-reflection rarely included acknowledgment of these stereotypes, indicating a discrepancy between implicit associations and declared attitudes. Participation in the association task elicited emotional responses, particularly surprise (54.5%), curiosity (42.4%), and discomfort (24.2%).

**Conclusions:**

These findings suggest that implicit cultural stereotypes may persist among nursing students despite explicit declarations of openness and professional values. These results highlight the limitations imposed by relying only on self-assessment in cultural competence education and indicate the potential value of approaches that engage unconscious cognitive processes through reflective and experiential learning. From the perspective of transformative learning theory, the emotional and reflective responses elicited by the projective task may support the development of critical self-awareness. Given the exploratory nature of this study and the limited sample size, these findings should be interpreted with caution and considered hypothesis generating.

## Highlights

• Unconscious cultural stereotypes were found among nursing students.
• Negative stereotypes dominated over positive and neutral associations.
• A clear gap existed between implicit bias and explicit self-reflection.
• Emotions like surprise and discomfort triggered reflective learning.
• Projective techniques proved useful in intercultural nursing education.

## الملخص

**الخلفية:**

تُعد الكفاءة الثقافية عنصراً أساسياً في الممارسة المهنية للتمريض، ولا سيما في بيئات الرعاية الصحية التي تتسم بالتنوع الثقافي والاجتماعي. وعلى الرغم من إظهار طلبة التمريض مواقف إيجابية تجاه التنوع والمساواة، فقد تستمر لديهم بعض الصور النمطية الثقافية غير الواعية التي قد تؤثر في تصوراتهم وتفاعلاتهم مع المرضى. ويصعب الكشف عن هذه الأنماط المعرفية الضمنية باستخدام أدوات التقييم الذاتي التقليدية وحدها. هدفت هذه الدراسة إلى استكشاف الصور النمطية الثقافية الضمنية التي تظهر من خلال التداعيات الذهنية التلقائية لدى طلبة التمريض، ودراسة التباين بين هذه التداعيات والتأمل الذاتي الواعي المتعلق بالكفاءة الثقافية في الممارسة التمريضية.

**طرق البحث:**

أُجريت دراسة استكشافية باستخدام المنهج المختلط، مع التركيز بصورة رئيسية على التحليل النوعي، في جامعة العلوم التطبيقية بمدينة كونين في بولندا خلال يونيو 2025. وشملت الدراسة 33 طالباً من طلبة الدراسات العليا في التمريض، اختيروا باستخدام أسلوب العينة الملائمة من أصل 41 طالباً دُعوا للمشاركة، بمعدل استجابة بلغ 80.49%. واستُخدم اختبار إسقاطي أُعد خصيصاً للدراسة، تضمن 14 جملة غير مكتملة صُممت لاستثارة الاستجابات التلقائية المرتبطة بالمواقف الثقافية والاجتماعية في بيئات الرعاية الصحية. وأعقب الاختبار نموذج للتأمل الذاتي تضمن سبعة أسئلة مفتوحة لاستكشاف الأنماط الفكرية والمشاعر المصاحبة للمهمة.

خضعت الاستجابات للتحليل الموضوعي النوعي باستخدام برنامج NVivo 14، وصُنفت إلى صور نمطية سلبية وإيجابية وعبارات واقعية محايدة. وأُجري تحليل وصفي كمي مكمل لتحديد التكرارات والنسب المئوية للاستجابات والانفعالات المبلغ عنها. وتولى باحثان مستقلان ترميز البيانات، وبلغ معامل اتفاق كوهين (Cohen’s Kappa) مقدار 0.85، مما يشير إلى مستوى مرتفع من الاتساق بين المقيمين.

**النتائج:**

شمل التحليل 482 وحدة استجابة، منها 174 استجابة تضمنت صوراً نمطية سلبية بنسبة 36.1%، و81 استجابة تضمنت صوراً نمطية إيجابية بنسبة 16.8%، في حين بلغت العبارات الواقعية المحايدة 227 استجابة بنسبة 47.1%. وتمحورت الصور النمطية بصورة رئيسية حول السلوك المتوقع من المرضى، والتواصل واللغة، والأصل القومي أو العرقي، والمعتقدات الدينية، والمظهر الخارجي، والوضع الاجتماعي والاقتصادي، والعلاقات الأسرية.

وأظهر التحليل النوعي أن بعض الاستجابات تضمنت افتراضات مسبقة تتعلق بسلوك المرضى أو قدرتهم على التواصل أو مدى تعاونهم مع مقدمي الرعاية الصحية، استناداً إلى خلفياتهم الثقافية أو الدينية. كما أظهرت بعض الاستجابات ربطاً بين محدودية إتقان اللغة البولندية وضعف الفهم، دون مراعاة العوامل السياقية الأخرى. وأشار تحليل التزامن اللفظي إلى وجود ارتباطات متكررة بين المفاهيم المتعلقة بسلوك المرضى وتوقعاتهم، والهوية الثقافية والدينية، وعمليات الرعاية الصحية.

وكشفت الدراسة عن تباين بين الاستجابات التلقائية والتأمل الذاتي الواعي؛ فعلى الرغم من ظهور محتوى نمطي في العديد من الاستجابات الإسقاطية، أقر مشارك واحد فقط صراحةً بوجود صور نمطية لديه خلال مرحلة التأمل الذاتي. كما أظهرت النتائج أن المشاركة في الاختبار أثارت مجموعة من الاستجابات الانفعالية، كان أبرزها الشعور بالمفاجأة بنسبة 54.5%، والفضول بنسبة 42.4%، وعدم الارتياح أو الإحراج بنسبة 24.2%، في حين بلغت نسبة اللامبالاة 6.1%، والمشاعر المختلطة أو غير المحددة 6.1%.

وأشارت هذه النتائج إلى أن التداعيات الذهنية التلقائية قد تكشف عن أنماط من التصورات الثقافية التي لا تظهر بالضرورة في التقييم الذاتي الواعي. كما أظهرت الاستجابات الانفعالية المصاحبة للاختبار إمكانية الاستفادة من التقنيات الإسقاطية في تحفيز التأمل النقدي والوعي الذاتي لدى طلبة التمريض.

**الاستنتاج:**

تشير نتائج هذه الدراسة إلى احتمال استمرار الصور النمطية الثقافية الضمنية لدى طلبة التمريض، حتى في ظل تبنيهم المعلن لقيم الانفتاح والمساواة والمهنية. وتبرز النتائج محدودية الاعتماد على التقييم الذاتي وحده في قياس الكفاءة الثقافية، وأهمية استكشاف الأساليب التعليمية التي تجمع بين التعلم التجريبي والتأمل النقدي لتعزيز الوعي بالتصورات الثقافية غير الواعية. وفي ضوء نظرية التعلم التحويلي، قد تسهم الاستجابات الانفعالية والتأملية التي تستثيرها التقنيات الإسقاطية في تطوير الوعي الذاتي النقدي لدى طلبة التمريض. ومع ذلك، فإن الطبيعة الاستكشافية للدراسة، وصغر حجم العينة، واقتصارها على مؤسسة تعليمية واحدة، تحد من إمكانية تعميم النتائج. ولذلك، ينبغي اعتبار هذه النتائج أولية ومولدة للفرضيات، مع الحاجة إلى دراسات مستقبلية أوسع نطاقاً للتحقق من فاعلية هذه الأساليب في تعزيز الكفاءة الثقافية وتحسين الممارسات التمريضية.

## Introduction

Cultural competence is widely recognized as a key element of high-quality nursing care and an essential prerequisite for effective communication, building trust, and ensuring patient safety in increasingly diverse healthcare systems.^1^ Nurses routinely interact with patients whose cultural backgrounds, beliefs, values, and health practices differ from their own, and thus cultural sensitivity is at the heart of professional nursing practice.^2^, ^3^ When cultural differences are not properly understood or interpreted using simplified cognitive schemas, they can negatively affect clinical assessment, interpersonal relationships, and the overall quality of care.^4^

A key barrier to providing culturally competent care is the presence of stereotypes. Stereotypes are generalized beliefs about individuals or social groups that reduce individual diversity to a limited set of attributed characteristics, often based on nationality, ethnicity, religion, gender, or appearance.^5^, ^6^, ^7^ Stereotypes can be positive or negative, and may sometimes appear neutral in tone, but they invariably function as cognitive shortcuts that shape the perception and interpretation of social reality.^8^ From a theoretical perspective, implicit stereotypes can be understood within dual-process models of cognition, which distinguish between automatic, unconscious processes and controlled, reflective reasoning.^6^, ^9^ In this framework, stereotypes are activated rapidly and without conscious intention, often preceding deliberate evaluation. This distinction is particularly relevant in healthcare contexts, where time pressure and cognitive load may increase reliance on automatic judgments.

In the healthcare environment, these simplifications can influence how patients are perceived, their behavior is interpreted, and professional decisions are made, sometimes without conscious reflection by medical staff.^10^, ^11^

Research indicates that stereotypes operate primarily at the implicit level, whereas explicit attitudes are consciously accessible and often aligned with socially desirable norms.^12^, ^13^, ^14^ In nursing and medical education, students often declare a high level of cultural awareness and openness, but empirical evidence suggests that unconscious cultural stereotypes may persist despite formal training and declarative knowledge.^15^, ^16^, ^17^ These implicit cognitive patterns are particularly difficult to identify using self-reporting tools because respondents may be unaware of their existence or reluctant to reveal socially undesirable associations.^18^, ^19^

The consequences of stereotypes in healthcare are well documented.^20^, ^21^, ^22^ Negative stereotypes can hinder communication, undermine trust, and contribute to unequal treatment of patients from minority or migrant groups. Although less overtly harmful, positive stereotypes can even limit a nurse's ability to see a patient as an individual with unique needs and experiences. These cognitive simplifications can have significant ethical and clinical implications in nursing practice where therapeutic relationships and holistic care are fundamental.

Previous studies conducted worldwide have demonstrated the persistence of implicit cultural stereotypes among nursing and medical students, highlighting discrepancies between self-assessed cultural competence and automatic cognitive responses.^23^, ^24^, ^25^, ^26^ These findings indicate a need for educational approaches that extend beyond knowledge transfer and explicitly address the unconscious dimensions of attitudes and beliefs.^27^, ^28^, ^29^, ^30^, ^31^ Projective techniques and experiential methods are increasingly recognized as valuable tools for identifying implicit cognitive patterns and supporting reflective learning in medical education.^32^, ^33^

Despite the growing number of global studies of stereotypes and implicit cultural stereotypes in healthcare, empirical data from Poland remain relatively limited.^34^, ^35^ The Polish socio-cultural context is undergoing dynamic changes related to migration and the growing diversity of patients, particularly due to recent geopolitical events. Intercultural education is formally included in nursing education programs but its practical effectiveness in reducing unconscious stereotypes is not clearly understood. This issue highlights the need for research that considers the local context and analyses of declared attitudes but also hidden cognitive patterns that may influence nursing practice. Previous studies mainly employed self-report or experimental measures, such as the Implicit Association Test (IAT), thereby hindering our understanding of how implicit stereotypes are expressed linguistically in context-specific healthcare scenarios.

The aims of this study were to explore unconscious cultural stereotypes manifested in the spontaneous associations of nursing students and to examine how these implicit cognitive patterns relate to students’ explicit self-reflection on cultural competence. The present study focused on stereotypes identified indirectly through language, emotions, and automatic associations rather than declared attitudes alone. By combining qualitative analysis of projective responses with a supplementary quantitative description of their frequency and emotional context, the aim was to provide a more comprehensive understanding of hidden cognitive mechanisms that may influence intercultural communication and nursing practice.

Accordingly, this study was guided by the following research question:

What types of unconscious cultural stereotypes are revealed in the spontaneous associations of nursing students, and how do these implicit associations differ from their explicit self-reflections regarding cultural competence in nursing practice?

## Materials and Methods

### Design

The study used a qualitative research design based on projective techniques. The study followed a QUAL → quan exploratory design, where quantitative data were used to support and contextualize qualitative findings rather than for inferential purposes. The quantitative component was embedded within the qualitative framework and served an illustrative rather than explanatory function. The research project was based on an interpretative paradigm assuming that meanings attributed to social reality, including relationships with patients, are constructed subjectively and may manifest themselves in unconscious linguistic reactions.^36^ The study was exploratory and diagnostic in nature, with an interpretivist epistemological stance, prioritizing the subjective meanings embedded in participants’ responses. The quantitative component was not intended to test hypotheses but to support the qualitative interpretation by illustrating the distribution and relative prominence of identified categories.

The choice of a projective approach was determined by the specific nature of unconscious cultural stereotypes, which are difficult to capture using traditional self-report tools. Standardized questionnaires or self-assessment scales primarily measure declared attitudes and conscious opinions, whereas stereotypes often function at an implicit and automatic level. In these cases, respondents may either be unaware of their biases or intentionally provide socially acceptable answers due to the social desirability effect.^19^

This approach is particularly valuable for research into cultural stereotypes in healthcare, where openly admitting bias may be rare or uncomfortable for participants.^37^

In the context of nursing education, projective techniques serve an educational and reflective function, where they can identify unconscious patterns but also provoke self-awareness and discussion about their professional implications. Thus, these techniques can be used as both a research and pedagogical tool, consistent with the interpretivist paradigm adopted in this study, which assumes that meanings and attitudes are socially constructed and may manifest indirectly through language.

### Sample and setting

The present study was conducted in June 2025 at the State University of Applied Sciences in Konin, Poland. Sample selection was based on convenience sampling. All students who met the inclusion criteria and were present at the University of Applied Sciences in Konin at the time of recruitment were invited to participate. The study participants were second-cycle nursing students. The inclusion criteria for the study were as follows: student status in the nursing program (second-cycle studies) and voluntary consent to participate in the study. The exclusion criterion was participation in the pilot study. Recruitment was carried out with the help of supervisors of individual year groups who were not involved in the study. Financial or academic incentives were not offered to respondents. Among the 41 students invited, 33 agreed to participate (response rate: 80.49%) (Table 1). The study involved 33 participants, which was considered sufficient to reach data saturation. During the analysis, no new themes or categories emerged after coding 33 responses, indicating that the data set was sufficiently rich to provide a comprehensive understanding of the subject under study.

**Table 1:** Respondent profiles.

Variable	Value
Age	Age range: 23–55 years
	Average age: 34 years
Place of residence	Town with up to 50,000 inhabitants: 6
	Town with 51,000–100,000 inhabitants: 15
	Town with over 100,000 inhabitants: 1
	Rural area: 11
Contact with patients from different cultures during practical training/work/everyday life?	Yes: 25
	No: 8
Participation in training on intercultural communication/cultural diversity?	Yes: 3
	No: 30
Self-assessment of knowledge about cultural differences	Very good: 1
	Good: 10
	Sufficient: 20
	Insufficient: 2

However, it should be acknowledged that the relatively small and homogeneous sample limits the transferability of the findings. In addition, the use of convenience sampling introduced potential selection bias because participants who volunteered may have been more interested in or sensitive to the issue of cultural competence.

### Study procedure

The original association test was constructed in several stages. First, a set of 18 incomplete sentences was developed based on a review of the literature and consultations with two experts in the field of intercultural communication and nursing. In order to assess the accuracy of the content, the experts evaluated each sentence in terms of its adequacy, clarity, and potential to evoke stereotypical associations. Their assessments confirmed the high accuracy of the tool.

The comprehensibility of the questions was tested in a pilot study involving 10 students from another university. Based on their feedback, four questions that caused difficulties were removed, leaving a final set of 14 sentences. The initial reliability of the tool was assessed by analyzing the consistency of response coding. Two independent coders who were trained in identifying stereotypes coded the responses from the pilot study. The coding consistency was measured as 0.85 using Cohen's Kappa coefficient, indicating the high reliability of the tool.

The study was conducted at the State University of Applied Sciences in Konin on June 2025. The participants were second-year nursing students who gave their voluntary consent to participate in the study. Recruitment was carried out through year supervisors, and participation in the study was not related to course grades or associated with any material or academic benefits.

The study was conducted in a university classroom after regular classes had ended. Before the study commenced, the researcher gave the participants verbal and written instructions, emphasizing that there were no “right” or “wrong” answers and that it was important to provide spontaneous and intuitive answers.

Completing all parts of the tool required 25–30 min on average. The researcher remained in the room throughout the study in order to provide explanations if necessary and to ensure that participants answered independently and under conditions of complete confidentiality.

### Instrument

An original Association Test of Cultural Stereotypes was designed specifically for this study due to the lack of available standardized Polish tools for assessing unconscious cultural associations among nursing students. The tool consisted of 14 incomplete sentences constructed according to projective principles (sentence completion technique). Each item referred to typical situations in healthcare settings that might evoke spontaneous cultural or social associations.

Participants were instructed to complete each sentence as quickly and spontaneously as possible, avoiding deliberate reflection or correction. After completing the association test, students filled in a self-reflection sheet, which contained seven open-ended questions that aimed to recognize their thought patterns and emotions during the task.

### Coding and data reliability

The responses were analyzed using inductive thematic coding supported by NVivo 14 (QSR International). In the first step, two independent researchers who were familiar with intercultural communication coded all of the responses according to emergent categories (appearance, behavior, religion, communication, family, and socio-economic status). In the second step, the codes were reviewed and merged into broader themes.

In total, 482 stereotypes were identified and grouped into three categories: negative stereotypes, positive stereotypes, and neutral facts. To ensure the reliability of coding, the inter-rater agreement was calculated using Cohen's Kappa to yield a coefficient of 0.85, indicating high consistency between coders. Discrepancies were discussed until full consensus was reached.

### Quantitative component

The study was primarily qualitative but supplementary quantitative analysis was performed to enhance the interpretation of the results. Descriptive statistics were used to determine the frequency and percentage distributions for responses classified into particular categories, i.e., positive, negative, and neutral stereotypes, as well as emotional reactions reported by participants. Each completed sentence in the association test was treated as a separate analytical unit. The frequency counts allowed the relative prevalence of different types of stereotypes and emotions to be assessed, thereby supporting the qualitative findings with numerical evidence. The quantitative component was exploratory in nature and served to illustrate the scale and proportion of identified phenomena rather than to test statistical hypotheses.

### Data interpretation strategy

The qualitative data were analyzed according to the principles of thematic analysis, as described by Braun and Clarke.^38^ This approach was selected due to its flexibility and suitability for identifying both semantic and latent meanings within textual data. The analysis proceeded through several iterative stages: (1) familiarization with the data by repeatedly reading all of the responses; (2) generating initial codes; (3) searching for recurring patterns and themes; (4) reviewing and refining themes in relation to the entire data set; (5) defining and naming the final themes; and (6) producing the analytic narrative supported by representative quotations.

During the interpretation process, particular attention was paid to both the explicit (manifest) content of students’ statements and the implicit (latent) meanings reflected in their emotional tone, choice of words, and spontaneous associations. This dual-level analysis was consistent with the projective nature of the instrument and facilitated the identification of hidden cognitive and emotional schemas underlying cultural stereotypes.^39^

## Results

### Overview of the findings

In total, 482 individual responses containing evaluative or generalizing content were identified in the association test and included in the analysis. Each completed sentence constituted a separate analytical unit. The results are presented in a structured manner, beginning with the response classifications, followed by analysis of the content of stereotypes, word co-occurrence patterns, emotional reactions to the task, and the discrepancy between implicit associations and explicit self-reflection. The results are presented in two complementary parts: quantitative overview of the response distribution and qualitative thematic analysis.

### Quantitative overview of response distribution

Students’ responses were classified into three categories according to their cognitive and semantic nature: negative stereotypes, positive stereotypes, and neutral factual statements (Table 2). Neutral responses constituted the largest proportion (47.10%) and included descriptive statements without evaluative judgment, such as references to nationality, language, religion, or observable characteristics.

**Table 2:** Classification of responses given in the association test in terms of their cognitive and semantic nature.

Category N = 482^a^	Stereotype		Neutral facts
	Positive	Negative	
Reponses	81 (16.80%)	174 (36.10%)	227 (47.10%)

a N refers to the total number of responses in answers rather than the number of participants.

Negative stereotypes predominated among the evaluative responses (36.10%), and positive stereotypes accounted for 16.80% of responses. Negative stereotypes most frequently involved generalizations related to patient behavior, perceived demands, hygiene, or trustworthiness. Positive stereotypes mainly concerned appearance or presumed personality traits. Although positive in tone, these responses also reflected simplified cognitive representations of patients.

### Content of cultural stereotypes: qualitative thematic findings

Qualitative thematic analysis showed that stereotypical associations most frequently concerned national or ethnic origin, religion, appearance, language and communication, socio-economic status, and family relationships. Assumptions related to nationality often included expectations regarding behavior or attitudes toward healthcare staff. Religious references were most commonly associated with Islam and visible religious symbols. Language-related stereotypes frequently linked limited proficiency in Polish with reduced understanding, without reference to situational or contextual factors.

Stereotypes were further classified according to their content, source, psychological function, and potential consequences for healthcare practice (Table 3). The identified psychological functions included explanatory, defensive, and identity related roles, and the potential consequences concerned communication difficulties, reduced trust, and the risk of unequal treatment.

**Table 3:** Classification of stereotypes according to content, source, psychological function, and consequences in healthcare.

Criterion	Category/dimension of stereotype
Stereotype content	• Appearance: “has a dark complexion,” “is handsome,” “dirty” • Character/personality traits: “is aggressive,” “sympathetic,” “is withdrawn” • Behaviors: “swears,” “complains about pain,” “talks a lot” • Religion and cultural practices: “he is a Muslim,” “he will pray,” “he does not shake hands” • Language and communication: “does not speak Polish,” “needs an interpreter” • Socio-economic status: “poor,” “rich grandfather,” “lives in poverty” • Family relationships: “has strong family ties,” “leaves the family”
Sources of stereotypes	• Ethnic/nationality (e.g., “A German is loud,” “a Ukrainian deserves everything”) • Religious (e.g., “A Muslim woman does not shake hands,” “A Hindu will pray”) • Linguistic/communicative (e.g., “he doesn't speak Polish, so he doesn't understand me”) • Socio-cultural (e.g., “Gypsy = thief,” “African woman = beautiful”) • Health/medical (e.g., “is infected,” “has COVID,” “has cancer”)
Psychological function	• Defensive stereotypes (protecting against anxiety, e.g., “problem patients”) • Explanatory stereotypes (simplify the world, e.g., “does not look into the eyes – lies”) • Identity stereotypes (reinforcing “us” vs. “them,” e.g., “Ukrainian – claimant”)
Consequences in healthcare	• Hindering communication and trust (e.g., “claimant patient”) • May lead to discrimination (e.g., “dirty,” “poor”) • Potentially supportive of the relationship (if used consciously, e.g., “family important in treatment” – included in care)

### Word co-occurrence analysis

Word co-occurrence analysis was conducted to explore the relationships between concepts appearing in students’ responses. The resulting network (Figure 1) shows that the strongest co-occurrences concerned concepts related to patient behavior and expectations, cultural or religious identity, and health or care processes. The sizes of the nodes in Figure 1 reflect the frequency of specific concepts, and the thicknesses of connections indicate the strength of their co-occurrence within the same responses.

**Figure 1:**
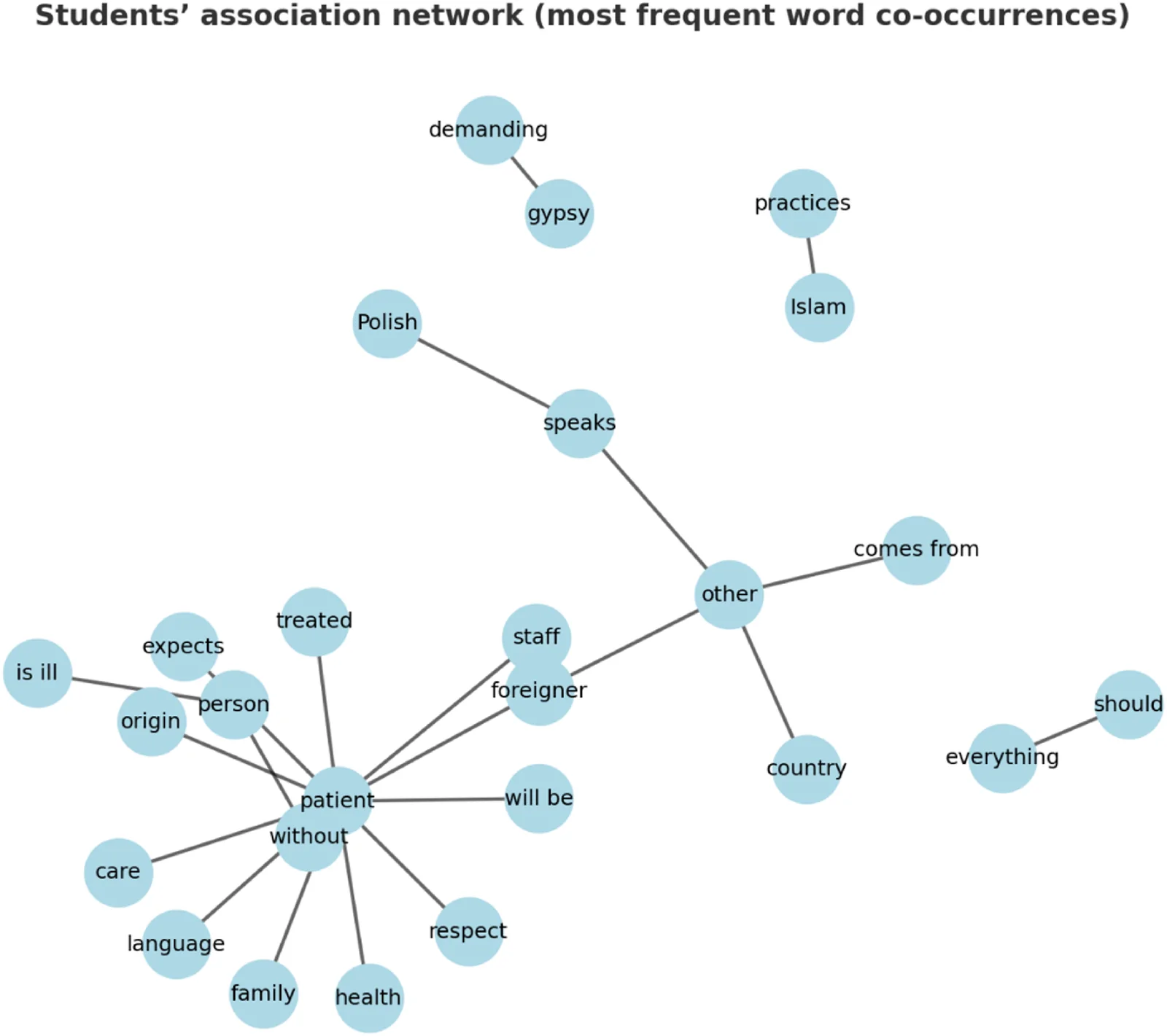
Map showing connections between most common words in students' answers.

### Emotional responses to the task

Participation in the association test elicited a range of emotional reactions (Table 4). The most frequently reported emotion was surprise (54.5%), followed by curiosity (42.4%) and discomfort or embarrassment (24.2%). Less frequently reported emotions included indifference (6.1%) and mixed or unspecified reactions (6.1%). These emotions were reported in the self-reflection sheet completed after the association task.

**Table 4:** Distribution of emotions and sample quotes from students.

Type of emotion	%	Sample quote
Surprise	54.5	“I was surprised that I automatically judged a patient by their appearance.”
Curiosity	42.4	“I want to know more about patients from other cultures.”
Discomfort/shame	24.2	“It was difficult for me when I noticed that I have a lot of stereotypes.”
Indifference	6.1	“This exercise did not show me anything new.”
Other/unspecified	6.1	“I had mixed feelings, and it's hard to name them.”

### Implicit–explicit discrepancy

A clear discrepancy was observed between students’ spontaneous associations and their explicit self-reflection. Numerous stereotypical associations were identified in the projective task, but only one participant explicitly acknowledged the presence of stereotypes in the self-reflection section. Figure 2 provides a conceptual illustration of the discrepancy between implicit associations revealed through spontaneous responses and explicit self-assessment. Figure 2 has an interpretative function and does not represent a quantitative comparison.

**Figure 2:**
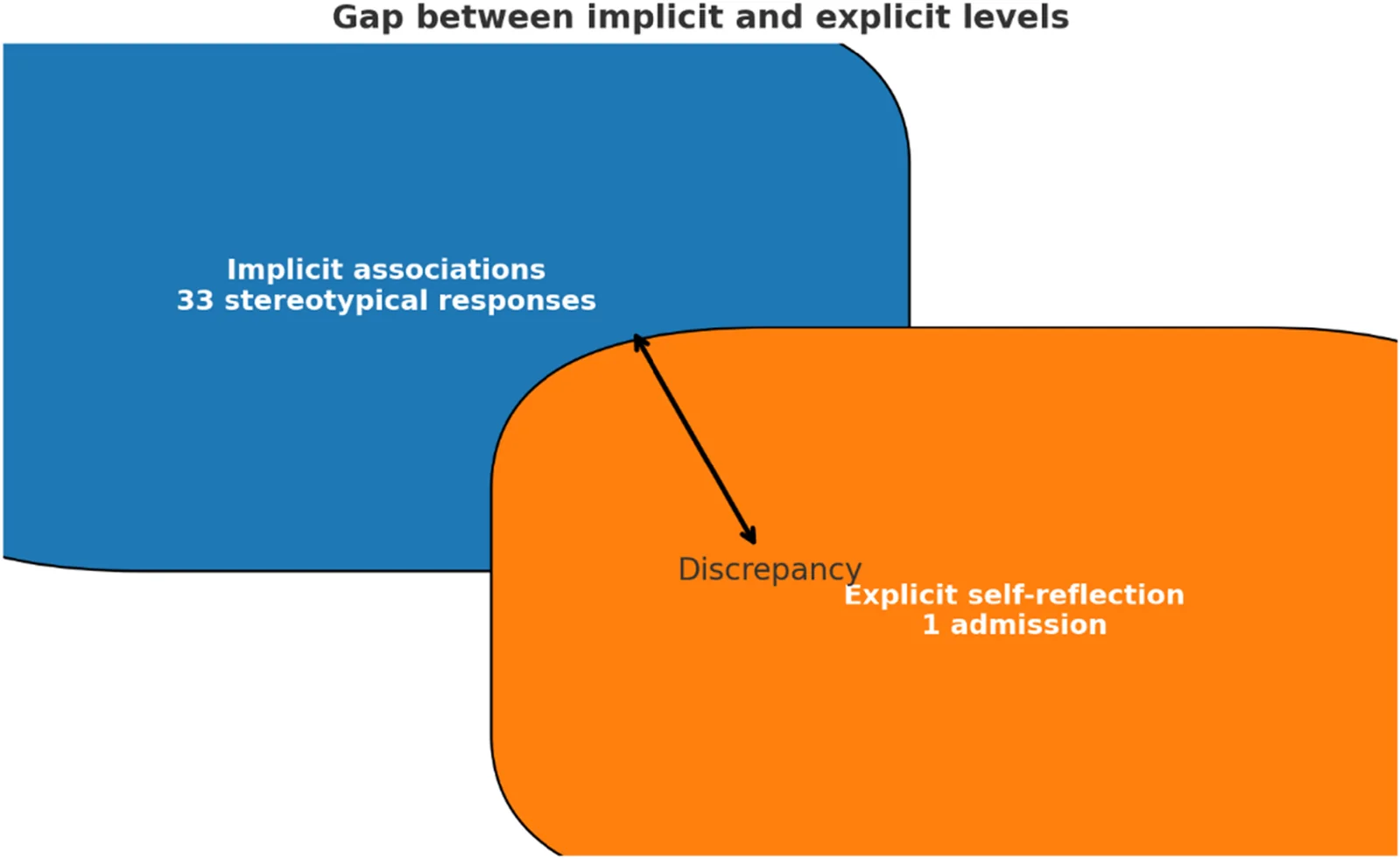
Gap between implicit associations and explicit self-reflection.

Note: The figure is an original conceptual illustration developed by the authors to illustrate the difference between students' unconscious (implicit) associations identified in the projective test and their conscious (explicit) self-assessment provided in the self-reflection sheet. The graphic does not represent quantitative data but serves an interpretative function by highlighting the cognitive and emotional gap between spontaneous linguistic reactions and declared self-awareness. The arrow marked “discrepancy” indicates that most participants revealed stereotypical content in their spontaneous responses but denied it in their self-description.

## Discussion

The present study explored unconscious cultural stereotypes among nursing students by analyzing their spontaneous associations and comparing them with explicit self-reflection. The findings indicated a discrepancy between the implicit cognitive patterns and declared attitudes, showing that implicit cultural stereotypes may persist despite formal education and conscious endorsement of egalitarian and professional values.^26^, ^40^ This observation is consistent with previous findings that implicit stereotypes often operate independently of explicit beliefs and may remain resistant to traditional educational approaches focused primarily on knowledge acquisition and self-assessment.

The predominance of negative stereotypes over positive stereotypes and neutral factual statements aligns with global studies of nursing and medical students.^24^, ^26^ Previous studies suggest that negative stereotypes tend to be more cognitively salient and emotionally charged, particularly in situations characterized by uncertainty, time pressure, or perceived interpersonal difficulty, such as intercultural healthcare encounters.^41^, ^42^ In the present study, negative stereotypes most frequently concerned patient behavior, communication, and social expectations, suggesting that anticipated interactional challenges may serve as key triggers for stereotypical thinking in clinical contexts.^43^, ^44^ However, these interpretations should be considered in light of the exploratory design and limited sample size.

Stereotypes related to national or ethnic origin and religion were especially prominent, with visible cultural markers functioning as automatic cues for categorization. Similar patterns have been reported in previous studies, indicating that observable differences such as appearance, language, or religious symbols may activate simplified explanatory frameworks used to manage cognitive load in complex professional environments.^45^, ^46^ These shortcuts may temporarily reduce uncertainty, but they risk oversimplifying patient experiences and undermining individualized, person-centered care.^6^, ^8^

An important finding obtained in this study was the limited awareness of stereotypes at the level of explicit self-reflection. Despite the frequent occurrence of stereotypical content in spontaneous associations, only one participant explicitly acknowledged the presence of stereotypes in their reflective responses. This discrepancy reflects a well-documented phenomenon in social cognition research,^15^, ^37^, ^47^ where individuals tend to perceive themselves as unbiased while primarily attributing prejudice to others. In nursing education, this gap may be reinforced by professional norms emphasizing ethical conduct and non-discrimination, which can unintentionally discourage the open acknowledgment of unconscious biases.^15^

The use of a projective association method was particularly effective for revealing these implicit cognitive patterns. Unlike self-report instruments, which primarily capture conscious attitudes and socially desirable responses, projective techniques elicit spontaneous linguistic reactions that provide insights into the content, emotional tone, and contextual meaning of implicit stereotypes. The situational framing of the association task within realistic healthcare contexts further enhances the validity of its real-world applicability and relevance to nursing practice.^48^, ^49^

The projective method differs fundamentally from the IAT, which is currently the most widely used tool for measuring implicit bias.^50^ The IAT relies on computer-based reaction times to infer the relative strengths of automatic associations, but it offers limited insights into the qualitative content or situational meaning of these associations. By contrast, the projective method facilitates direct analysis of how implicit stereotypes are expressed through language in professional scenarios familiar to nursing students. Moreover, the results produced by the IAT are typically presented as abstract numerical scores,^51^ whereas projective responses can be directly integrated into reflective discussions, supervision, and educational feedback.^52^ Thus, from an educational perspective, the projective method is particularly suitable for fostering self-awareness and reflective learning rather than only diagnosing implicit bias.^53^

The emotional responses elicited by the association task provide further insights into its educational potential, particularly surprise and discomfort. These reactions suggest moments of cognitive dissonance triggered by confrontation with automatic associations that conflicted with the students’ self-perceptions as culturally open and unbiased professionals. Similar emotionally challenging experiences have been identified as important catalysts for reflective learning and professional development.^54^, ^55^, ^56^

These findings can be interpreted through the lens of Mezirow's transformative learning theory. From this perspective, the projective association task appeared to initiate the early phases of perspective transformation. The reported emotions of surprise and discomfort correspond to a *disorienting dilemma*, defined as an experience that challenges previously taken-for-granted assumptions.^56^ In the present study, this moment occurred immediately after completing the association task when students were confronted with the discrepancy between their spontaneous responses and declared values.

The transition from a *disorienting dilemma* to *critical assessment of assumptions* was evident during the subsequent self-reflection phase. The explicit acknowledgment of stereotypes was rare, but the expressed emotional reactions indicate an emerging awareness that automatic associations did not fully align with professional ideals, suggesting the beginning of critical examination of internalized cognitive schemas, particularly those related to patient behavior, communication styles, and cultural or religious identity. In addition, the limited articulation of stereotypes in reflective writing indicates that many students remained in an early stage of transformation where emotional dissonance had been triggered but not yet translated into the conscious reframing of meaning perspectives.

From an educational standpoint, these findings highlight the importance of structured support for facilitating further progression through the transformative learning process. Guided reflection, facilitated discussion, or debriefing sessions may help students move beyond initial discomfort toward deeper critical analysis, and integrating more inclusive frames of reference into professional practice. Thus, projective and reflective techniques are valuable tools for addressing implicit cognitive processes that are often overlooked in traditional intercultural education focused on declarative knowledge and ethical norms.

### Strengths and limitations

A key strength of this study was its focus on unconscious cognitive processes, which are rarely examined using indirect methods in nursing education research. The combination of qualitative thematic analysis with supplementary quantitative description facilitated more nuanced exploration of the content and distribution of stereotypes. In addition, the use of projective techniques facilitated access to automatic associations that may not be captured through self-report instruments.

Several limitations must also be acknowledged. The present study was conducted at a single institution using convenience sampling, limiting the generalizability of the findings. The relatively small sample size and specific socio-cultural context may also have influenced the nature of the stereotypes identified. Furthermore, efforts were made to ensure analytical rigor through independent coding and high inter-rater reliability, but qualitative interpretation remains inherently subjective. Future research could expand on these findings by including longitudinal designs, larger and more diverse samples, and interventions aimed at reducing implicit cultural stereotypes.

## Conclusions

This study was exploratory in nature and the findings obtained should be interpreted as hypothesis generating rather than definitive.

The findings obtained in this study suggest that unconscious cultural stereotypes may be present among nursing students and persist independently of explicit declarations of openness and professional values. Students tended to describe themselves as culturally sensitive at the conscious level, but spontaneous associations revealed a predominance of negative stereotypical content, particularly in relation to patient behavior, communication, and social expectations. This discrepancy may point to the limitations of relying solely on self-assessment tools when evaluating cultural competence in nursing education.

The results suggest that educational approaches focused exclusively on declarative knowledge and ethical norms may be insufficient for addressing implicit cognitive processes that influence clinical perception and interaction. Projective methods combined with guided self-reflection are promising approaches for identifying unconscious stereotypes and initiating critical awareness in a safe educational context. From the perspective of transformative learning theory, these methods may support the early stages of perspective transformation by confronting students with discrepancies between their implicit assumptions and professional ideals.

Importantly, these findings also have practical implications for nursing education. The results obtained from projective association tests may be effectively used for structured debriefing following high-fidelity medical simulations. Integrating students’ own spontaneous associations into post-simulation reflection may help link their technical performance with deeper analysis of communication, attitudes, and implicit assumptions toward culturally diverse patients. Thus, projective techniques can complement simulation-based education by addressing procedural skills but also the cognitive and affective dimensions of professional competence.

Overall, this study suggests the potential value of incorporating reflective and experiential strategies that explicitly engage implicit cultural stereotypes into nursing curricula, thereby better preparing future nurses for equitable, individualized care in increasingly diverse healthcare settings. However, these results should be interpreted as hypothesis generating and require confirmation in larger and more diverse samples.

## Author contribution

Małgorzata Lesińska-Sawicka: Writing – review & editing, Writing – original draft, Project administration, Methodology, Investigation, Formal analysis, Data curation, Conceptualization.

## Ethical declaration

The required approval from the Ethics Committee was obtained before the study was conducted at the University of Applied Sciences in Konin (Resolution NO 2 Committee on Ethics in Scientific Research University of Applied Sciences in Konin, dated June 24, 2025). Respondents were informed that they could withdraw from the study without giving any reasons and without any consequences; that their responses would be anonymized by removing any personal information, and would be analyzed with other responses to obtain aggregate results; no identifying information would be included in this data set; and there was no direct personal benefit associated with participation in this study.

## Statement for ethical approval

Author declare that she has obtained approval from the Bioethics Committee to conduct the study - Resolution NO 2 Committee on Ethics in Scientific Research University of Applied Sciences in Konin dated 24 June 2025.

## Source of funding

This study did not receive any specific grant from funding agencies in the public, commercial, or not-for-profit sectors.

## Conflict of interest

Author declare that she has no conflicts of interest related to the content of this manuscript. No financial, personal, or professional affiliations or relationships influenced the research, analysis, or conclusions presented in this study.
